# Rising Rates of Hepatocellular Carcinoma Leading to Liver Transplantation in Baby Boomer Generation with Chronic Hepatitis C, Alcohol Liver Disease, and Nonalcoholic Steatohepatitis-Related Liver Disease

**DOI:** 10.3390/diseases5040020

**Published:** 2017-09-26

**Authors:** George Cholankeril, Eric R. Yoo, Ryan B. Perumpail, Andy Liu, Jeevin S. Sandhu, Satheesh Nair, Menghan Hu, Aijaz Ahmed

**Affiliations:** 1 Division of Gastroenterology and Hepatology, Stanford University School of Medicine, Stanford, CA 94305, USA; gcholankeril@gmail.com (G.C.); jsandhu@oxy.edu (J.S.S.); 2 Department of Medicine, Santa Clara Valley Medical Center, San Jose, CA 95128, USA; eric.r.yoo@gmail.com; 3 Division of Gastroenterology and Hepatology, University of California, Los Angeles, CA 90095, USA; rperumpail@gmail.com; 4 Department of Medicine, California Pacific Medical Center, San Francisco, CA 94114, USA; andyeliu@gmail.com; 5 Division of Gastroenterology and Hepatology, University of Tennessee Health Sciences Center, Memphis, TN 38163, USA; snair@uthsc.edu; 6 Department of Biostatistics, Brown University School of Public Health, Providence, RI 02912, USA; menghan_hu@brown.edu

**Keywords:** baby boomer, hepatitis C virus, alcoholic liver disease, non-alcoholic steatohepatitis, liver transplantation

## Abstract

We aim to study the impact of the baby boomer (BB) generation, a birth-specific cohort (born 1945–1965) on hepatocellular carcinoma (HCC)-related liver transplantation (LT) in patients with chronic hepatitis C virus (HCV), alcoholic liver disease (ALD), and non-alcoholic steatohepatitis (NASH). We performed a retrospective analysis using the United Network for Organ Sharing (UNOS)/Organ Procurement Transplant Network (OPTN) database from 2003 to 2014 to compare HCC-related liver transplant surgery trends between two cohorts—the BB and non-BB—with a secondary diagnosis of HCV, ALD, or NASH. From 2003–2014, there were a total of 8313 liver transplant recipients for the indication of HCC secondary to HCV, ALD, or NASH. Of the total, 6658 (80.1%) HCC-related liver transplant recipients were BB. The number of liver transplant surgeries for the indication of HCC increased significantly in NASH (+1327%), HCV (+382%), and ALD (+286%) during the study period. The proportion of BB who underwent LT for HCC was the highest in HCV (84.7%), followed by NASH (70.3%) and ALD (64.7%). The recommendations for birth-cohort specific HCV screening stemmed from a greater understanding of the high prevalence of chronic HCV and HCV-related HCC within BB. The rising number of HCC-related LT among BB with ALD and NASH suggests the need for increased awareness and improved preventative screening/surveillance measures within NASH and ALD cohorts as well.

## 1. Introduction

The incidence of hepatocellular carcinoma (HCC) has been rising at an alarming rate with over half a million new cases diagnosed annually worldwide [1]. In the United States (U.S.), HCC is the most rapidly rising cause of cancer and cancer-related deaths with an incidence that has tripled over the last decade [2]. This rise in HCC incidence is largely due to the high prevalence of chronic hepatitis C virus (HCV) infection, which has recently surpassed 3 million people [3]. 

The incidence of HCV has shown a disproportionate birth specific-cohort effect, with up to 80% of HCV infection seen in baby boomers (BB), Americans born between 1945 and 1965 [4]. Due to their long-standing chronic HCV infection, this aging birth specific-cohort has a higher likelihood for developing HCC [5,6]. With an otherwise dismal prognosis, select HCC patients may be candidates for liver transplantation (LT). The number of HCV-infected BB with HCC awaiting LT has already increased nearly four-fold over the last two decades [7,8]. However, the impact of HCC LT among BB within other leading liver disease etiologies, such as non-alcoholic steatohepatitis (NASH) and alcoholic liver disease (ALD), has yet to be determined. We aim to study the impact of the BB birth-specific cohort on LT recipients with HCV-related HCC, ALD-related HCC, and NASH-related HCC.

## 2. Methods

We utilized the registry data from the United Network for Organ Sharing (UNOS)/Organ Procurement Transplant Network (OPTN) database to compare HCC LT trends in all adults (age ≥ 18) from 2003 to 2014. Birth cohort-specific disparities in HCC LT were evaluated by categorizing patients into two cohorts: BB and non-BB (non-baby boomers—born pre-1945 or post-1965). The underlying etiology of HCC was determined by secondary diagnosis coding of liver disease etiology among patients with HCC undergoing LT. Liver disease etiologies were categorized into HCV, ALD, or NASH. In addition to using patients listed with a secondary diagnosis of NASH with HCC, we estimated the number of patients with NASH in cryptogenic cirrhosis or cirrhosis due to unknown etiology categories on a body mass index based on previously defined criteria [8]. 

We studied the proportion of HCC-related liver transplant surgeries according to their secondary etiology and analyzed the annual trends in these breakdowns from 2003 and 2014. Additionally, we compared demographic data (age, gender, ethnicity), and clinical comorbidities including hepatic encephalopathy (HE), ascites, and diabetes within these two cohorts using chi-square testing for categorical variables. Statistical significance was met using a two-tailed *p* value < 0.05. Post-transplant survival was analyzed with Kaplan–Meier methods. All statistical analyses were performed with Stata (Version 10; Stata Corporation, College Station, TX, USA).

## 3. Results

From 2003 to 2014, there were a total of 8313 HCC-related liver transplant surgeries for HCC secondary to HCV, ALD, or NASH. HCV-related HCC had the highest proportion of LT (*n* = 6034, 72.6%) followed by NASH-related HCC (*n* = 1350, 16.2%) and ALD- related HCC (*n* = 929, 11.2%). The BB cohort constituted 6658 (80.1%) of all HCC-related liver transplant recipients. From 2003 to 2014 the number of HCC-related liver transplant recipients increased nine-fold in NASH (+905%) and more than doubled in HCV (+268%) and ALD (+208%). In a similar fashion, the number of liver transplant surgeries for HCC among BB rose significantly in NASH (+1327%), HCV (+382%), and ALD (+286%). The overall annual trends in HCC-related LT among BB for HCV, NASH, and ALD are depicted in Table 1. The proportion of BB who underwent LT for HCC was the highest in HCV (84.7%), followed by NASH (70.3%), and ALD (64.7%). The highest annual increment in BB proportion of HCC-related LT was noted in NASH (+3.5%), followed by HCV (+2.6%), and ALD (+2.1%).

Demographic and clinical characteristics between BB and non-BB cohorts undergoing LT for HCC among HCV, NASH, and ALD are outlined in Table 2. Compared to non-BB, HCC-related LT in BB also had a higher prevalence of males in HCV (BB, 80.3% vs. non-BB, 66.1%, *p* < 0.01) and NASH (BB, 73.0% vs. non-BB, 65.1%, *p* < 0.01) (Table 2). The BB HCC cohort also had a higher prevalence of other complications of end-stage liver disease including HE (BB, 40.2% vs. non- BB, 34.8%, *p* < 0.01) and ascites (BB, 50.9% vs. non-BB, 46.8%, *p* < 0.01). 

**Figure 1:**
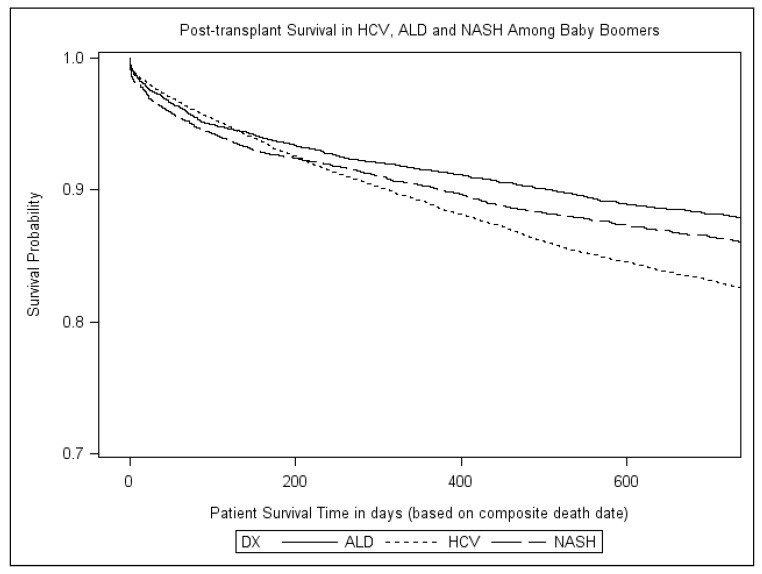
Kaplan–Meier Survival Curves in HCC Post-Liver Transplantation among Baby Boomers with HCV, ALD, and NASH.

There was no statistical difference (*p* > 0.05) in short-term (one-year) post-transplant survival rate among BB vs. non-BB HCC-related LT in HCV (BB, 76.1% vs. non-BB, 77.2%), NASH (BB, 76.0% vs. non-BB, 75.6%), or ALD (BB, 77.4% vs. non-BB, 79.0%). However, when comparing post-transplant survival in BB within the three liver disease etiologies, post-transplant survival was highest in ALD, followed by NASH and HCV (Figure 1). 

## 4. Discussion

Since the implementation of the Model for End Stage Liver Disease (MELD) for liver allocation, the number of HCC patients who underwent LT has risen significantly. While previous studies have attributed this rising trend in HCC incidence and surgical intervention to BB cohort, these studies only evaluated its impact on HCV-related HCC [9]. Although HCV continues to be the leading liver disease etiology for HCC-related LT, there is a significant rise in NASH-related HCC and ALD-related HCC leading to LT. Our analysis suggests that the BB cohort has influenced the rise in overall HCC-related LT in all three liver disease etiologies. Aside from the birth specific-cohort effect, our current allocation policy has also influenced the dramatic rise in the number HCC-related LT. Candidates with HCC awaiting LT are eligible to receive a MELD exception resulting in higher priority and increased likelihood of undergoing liver transplant surgery [10].

As the BB generation age, they have an increased risk of developing and suffering from chronic health conditions and present a complicated challenge for healthcare providers in the future. Therefore, there will be an increasing demand to allocate resources on policies highlighting preventative health in the BB cohort. The 2012 guideline set by the Centers for Disease Control and Prevention, the 2013 guideline set by the United States Preventative Services Task Force (USPSTF), and the 2014 guideline set by the American Association for the Study of Liver Disease (AASLD) in conjunction with the Infectious Disease Society of America have highlighted the increasing evidence of the benefits of age-based HCV testing [1,11]. The recommendations for birth-cohort specific HCV screening stemmed from a greater understanding of the high prevalence of chronic HCV and HCV-related HCC within the BB generation. However, the rising number of HCC-related LT among BB generation with ALD and NASH suggests the need for increased awareness and improved preventative screening/surveillance for HCC in these sub-cohorts.

## Figures and Tables

**Table 1:** Baby Boomer HCC Liver Transplantation Annual Trends with HCV, ALD, and NASH; UNOS 2003–2014.

	HCV			NASH			ALD		
	BB ^†^	Overall	Percent	BB	Overall	Percent	BB	Overall	Percent
2003	149	216	69.0%	11	19	57.9%	22	37	59.5%
2004	167	224	74.6%	20	38	52.6%	16	33	48.5%
2005	218	297	73.4%	22	47	46.8%	28	59	47.5%
2006	271	351	77.2%	37	71	52.1%	36	75	48.0%
2007	373	461	80.9%	54	90	60.0%	38	85	44.7%
2008	427	505	84.6%	80	124	64.5%	48	79	60.8%
2009	460	540	85.2%	96	151	63.6%	56	76	73.7%
2010	492	585	84.1%	101	132	76.5%	45	66	68.2%
2011	567	632	89.7%	91	128	71.1%	81	107	75.7%
2012	619	707	87.6%	127	168	75.6%	77	106	72.6%
2013	646	722	89.5%	153	191	80.1%	69	92	75.0%
2014	719	794	90.6%	157	191	82.2%	85	114	74.6%
Total	5108	6034	84.7%	949	1350	70.3%	601	929	64.7%
APC ^‡^			+2.6%			+3.5%			+2.1%

^†^ BB = Baby Boomer; ^‡^ APC = Annual Percent Change.

**Table 2:** Demographic and Clinical Characteristics in HCC Liver Transplant Recipients among Baby Boomers versus Non-Baby Boomers; UNOS 2003–2014.

	HCV			NASH			ALD		
	BB *n* = 5108	Non-BB *n* = 926	*p*	BB *n* = 949	Non-BB *n* = 401	*p*	BB *n* = 601	Non-BB *n* = 328	*p*
Age, median	57	66	<0.01	59	67	<0.01	58	67	<0.01
Gender									
Male	80.3%	66.1%	<0.01	73.0%	65.1%	<0.01	90.5%	89.6%	0.67
Ethnicity									
White	67.7%	59.1%	<0.01	75.9%	77.6%	0.50	69.6%	78.7%	<0.01
Black	13.3%	11.1%	0.07	5.4%	3.7%	0.20	3.8%	1.2%	0.02
Hispanic	13.6%	17.6%	<0.01	14.7%	14.5%	0.93	14.7%	14.5%	0.14
Asian	4.1%	11.1%	<0.01	2.6%	2.2%	0.67	3.0%	1.2%	0.89
Other	9.9%	1.1%	<0.01	1.4%	2.0%	0.49	8.9%	4.4%	<0.05
HE	40.2%	34.8%	<0.01	43.4%	41.4%	<0.50	50.3%	43.9%	0.09
Diabetes	23.6%	28.4%	<0.01	47.7%	47.7%	<0.01	35.1%	33.5%	0.63
Ascites	50.9%	46.8%	<0.05	55.2%	55.2%	0.84	68.6%	62.5%	0.06
